# How to recognize a trustworthy clinical practice guideline

**DOI:** 10.1186/s44158-023-00094-7

**Published:** 2023-04-28

**Authors:** João Pedro Lima, Reza D. Mirza, Gordon H. Guyatt

**Affiliations:** Department of Health Research Methods, Evidence, and Impact, McMaster University, 1280 Main Street West 2C Area, Hamilton, ON L8S 4K1 Canada

**Keywords:** Clinical practice guidelines, GRADE approach, Evidence-based medicine, Recommendation

## Abstract

Trustworthy clinical practice guidelines represent a fundamental tool to summarize relevant evidence regarding a set of clinical choices and provide guidance for making optimal clinical decisions. Clinicians must differentiate between guidelines that provide trustworthy evidence guidance and those that do not. We present six questions clinicians should ask when evaluating a guideline’s trustworthiness. (1) Are the recommendations clear?; (2) Have the panelists considered all alternatives?; (3) Have the panelists considered all patient-important outcomes?; (4) Is the recommendation based on an up-to-date systematic review?; (5) Is the strength of the recommendation compatible with the certainty of the evidence?; (6) Might conflicts of interest influence the recommendations? If yes, were they managed? Once the conclude they are dealing with a trustworthy guideline, clinicians must gain an understanding of the transparent evidence summary that the guideline will offer, and judge the applicability of trustworthy recommendations to their patients and settings. Consideration of the circumstances and values and preferences of patients will be crucial for all weak or conditional recommendations.

## Background

The practice of evidence-based medicine presents challenges. Clinicians typically may not have the skills or the time to review primary studies, or even systematic reviews, to determine their rigor and carefully consider their results and implications for practice [1, 2].

To address this issue, trustworthy clinical practice guidelines should serve as a fundamental tool to summarize the evidence and provide guidance for clinical decision-making. Guidelines may, however, be well or poorly conducted and, if poorly conducted, offer guidance that is not in patients’ best interests. Thus, it is incumbent on clinicians to differentiate between guidelines that are trustworthy from those that are not.

Guidelines remain inconsistent in their development, reporting, and management of conflicts. Indeed evaluations of guideline rigor using existing checklists (AGREE I and II [3, 4], and the Neat instrument based on criteria from the Institute of Medicine [5]) have demonstrated that although recent years have seen improvement, guidelines continue to frequently suffer from major limitations [6–9]. Those limitations include failure to base recommendations on systematic reviews, failure to adequately address conflict of interest, and neglect of values and preferences*.* Given the limitations of existing guidelines, users require an approach to recognize trustworthy practice guidelines.

## How to recognize a trustworthy clinical practice guideline

Building on prior User’s Guides to the Medical Literature addressing guidelines [10–12], we present six questions (Table 1) clinicians should ask when evaluating a guideline’s trustworthiness and a final section regarding the applicability of the guideline to their clinical setting and patients.

**Table 1:** Six questions to assess a guideline’s trustworthiness

(1) Are the recommendations clear?
(2) Have the panelists considered all alternatives?
(3) Have the panelists considered all patient-important outcomes?
(4) Is the recommendation based on an up-to-date systematic review?
(5) Is the strength of the recommendation compatible with the certainty of the evidence?
(6) Might conflicts of interest influence the recommendations? If yes, were they managed?

1. *Are the recommendations clear?*
  Clinical Practice Guidelines should provide clear and actionable recommendations [13]. To achieve clarity, guidelines must state the direction (i.e., in favor or against) and strength (i.e., strong or weak/conditional) of their recommendations [12]. To be actionable, recommendations should define the context in which the interventions are recommended, including patient population and setting.
  For instance, in a living guideline on drugs for COVID-19 [14], the authors “recommend treatment with systemic corticosteroids (strong recommendation)” for patients with severe or critical COVID-19. Elsewhere, they “recommend not to use lopinavir-ritonavir (strong recommendation against)” regardless of disease severity. In both cases, the authors have made clear the population and intervention; the strength of the recommendations. The comparator – standard care without corticosteroids or lopinavir-ritonavir — while implicit, is evident.
  Guidelines may, however, be unclear and, therefore, difficult to interpret. Jin YH et al., 2020 [15] make a recommendation in favor of remdesivir for patients with COVID-19 without specifying the severity of the patient population (e.g., mild to moderate, severe, or critical). Clear recommendations should make clear the patient population, intervention, and the comparator being addressed.
2. *Have the panelists considered all alternatives?*
  When making a recommendation, guidelines should address all alternatives that physicians might consider [12]. Comparators may be standard of care or other interventions. Although studies may have compared interventions to a placebo, because clinicians do not consider placebos in providing care to patients, they are not appropriate comparators in practice guidelines.
  The National Institute for Health and Care Excellence (NICE) [16] recommends the use of vasoactive drugs for pediatric septic shock. However, there are no recommendations comparing one vasopressor over others, leaving clinicians uncertain which agent to choose. On the other hand, the Surviving Sepsis Campaign International Guidelines for the Management of Septic Shock and Sepsis-Associated Organ Dysfunction in Children [17] not only recommends the use of those vasoactive drugs but also prioritizes the administration of noradrenaline and adrenaline over dopamine in children with septic shock. In summary, guidelines that explicitly address the complete range of alternatives that clinicians may consider will be more useful than those that do not.
3. *Have the panelists considered all patient-important outcomes?*
  While clinical trials often focus on primary outcomes, patients choosing between alternatives are typically interested in a number of consequences that will ensue depending on their choice. These may include mortality and major morbid events such as stroke, and outcomes related to quality of life, such as function and pain, typically measured using patient-reported outcome measures. Trustworthy guidelines must consider all patient-important outcomes, including both benefits and harms.
  What guidelines should not focus on is outcomes, such as hypoxemia, a physiology score, or cardiac output, that may be biologically compelling but are not in themselves important to patients. We call such outcomes “surrogate” or “substitute” endpoints that act as stand-ins for what is important to patients. To distinguish between a surrogate and a patient-important outcome one can ask oneself the following question: if the outcome under consideration were the only one to improve with treatment, would the patient be interested in using a treatment associated with harms and burdens? Patients told that a treatment that improves their oxygenation, their physiology score, or increases cardiac output but does not prolong their lives, prevent major morbid events, make them feel better, or shorten their stay in a critical care unit would not be interested. Oxygenation, physiology score, or cardiac output are therefore surrogate outcomes. In contrast, patient-reported outcomes such as breathlessness or quality of life are important to patients and their treating physicians, are often the reason for their presentation and often do show low correlations with surrogates.
  Why would guideline developers be tempted to rely on such surrogates? Clinical trialists often focus on surrogate laboratory markers and imaging results because they can conduct much shorter trials with many fewer patients than would be required to detect effects on mortal or major morbid outcomes. Indeed, surrogates may be all that existing trials have addressed.
  In such instances, guidelines should specify the patient-important outcome for which the surrogate outcome is standing in and acknowledge that indirect evidence leaves uncertain the impact of the intervention on the corresponding patient-important outcome. A treatment that increases cardiac output may or may not improve function and reduce hospitalizations, and one that improves oxygenation may or may not reduce mortality in patients with acute respiratory distress syndrome (ARDS).
  Guidelines must consider both benefits and harms. The importance of harms is likely relative to the harm presented by the clinical condition and thereby the potential for benefit for mitigating harm. While in life-threatening circumstances, such as necrotizing fasciitis, patients tend to accept higher risks of treatments such as emergency fasciotomy, risk tolerance will be lower in chronic diseases in which benefit is likely to be more modest.
  The Intensive Care Society has issued recommendations suggesting the use of subglottic secretion drainage to reduce ventilator-associated pneumonia (VAP), duration of mechanical ventilation, and length of the ICU stay. In making their recommendation, they make no reference to complications related to the procedure [18] that include transient dyspnea, upper airway obstruction, and dysphonia at extubation [19]. Clinicians will be appropriately skeptical of guidelines that omit consideration of important harms of burdens.
4. *Is the recommendation based on an up-to-date systematic review?*
  New evidence may differ from prior study results; thus, recommendations may also change. Recombinant activated protein C in septic shock provides an example of such an evidence shift. Activated protein C, once promoted for septic shock [20], ultimately demonstrated no reduction in the risk of death and an increase in the risk of bleeding [21]. If new practice-changing is available, recent guidelines will be more credible than previous ones.
  For example, the 2021 European Society of Cardiology (ESC) Guidelines [22] for the diagnosis and treatment of acute and chronic heart failure issued recommendations related to the treatment of heart failure with preserved ejection fraction (HFpEF). In this guideline, authors do not recommend sodium-glucose cotransporter-2 (SGLT2) Inhibitors for patients with HFpEF. Clinical trials published very shortly after the authors’ deadline for new evidence have provided high certainty evidence that SGLT2 Inhibitors reduce hospitalization in such patients [23, 24]. As of February 2023, this guideline, omitting this crucial therapy for patients with HFpEF, still represented the recommendations of the ESC. This highlights the need for clinicians to use updated guidelines that reflect the current best evidence.
5. *Is the strength of the recommendation compatible with the certainty of the evidence?*
  Recommendations may be strong (right for all, just do it) versus weak or conditional (right for the majority but not all, consider the circumstances). The GRADE approach [25], endorsed by over 110 guideline organizations worldwide, represents the existing standard for both rating the certainty (synonymous with quality) of evidence and also grading the strength of recommendations. GRADE rates the certainty of a body of evidence as high, moderate, low, or very low; randomized trials start as high, observational studies as low, with further considerations of risk of bias, imprecision, inconsistency, indirectness, and publication bias.
  In the GRADE formulation, a panel issues strong recommendations when benefits clearly outweigh downsides — or the reverse. When the balance is less certain, panels issue weak recommendations. When clinicians see a strong recommendation, they can infer that all or almost all fully informed individuals would choose the same treatment option; when they see a weak or conditional recommendation they can infer that the majority of informed patients would choose the recommended option, but a minority, typically because of different values and preferences, would not.
  In general, guideline panels should not issue strong recommendations in the face of low certainty evidence: if one is uncertain of the benefits, harms, and burdens of a treatment, it is very unlikely that one will be confident that the benefits outweigh the downsides, or the reverse. There are, however, exceptional circumstances when a panel may reasonably make a strong recommendation in the face of low certainty evidence. These include (1) life-threatening conditions, (2) uncertain benefit with certain harm, (3) options equivalent in benefits with one being less harmful or costly, and (4) potential catastrophic harm [12]. Generally, however, clinicians should view a strong recommendation for an intervention in the face of low certainty evidence as a red flag for a possible untrustworthy guideline.
  Detecting such a problem — indeed, making any judgment of whether the evidence warrants the panel’s recommendation — requires a transparent and easily understandable presentation of the evidence, including absolute estimates of benefits and harms of the interventions. GRADE suggests including summary-of-findings (SoF) tables [26–28] to achieve such presentations. SoF tables provide clinicians and patients with relative and absolute estimates along with the certainty of evidence for each outcome. In that way, information becomes more digestible to both.
  In Fig. 1, we see an example of a SoF table from the World Health Organization’s living COVID-19 guideline addressing baricitinib in patients with critical or severe illness. The first column lists outcomes, starting with mortality. The second column reports the relative effect estimate (in this case an odds ratio) for each outcome, with the associated 95% confidence interval and the number of patients, studies, and types of studies meta-analyzed. In this case, estimated odds of mortality decreases by 17% based on 10,815 patients across 4 randomized controlled trials with baricitinib compared to standard of care. The next two columns provide absolute estimates of the outcome: 110 deaths per 1000 patients in those treated with baricitinib, compared with 130 per 1000 with standard of care, a difference of 20 fewer per 1000 with a 95% confidence interval of 30 fewer to 8 fewer. The penultimate column presents the certainty of evidence as assessed by GRADE, high quality for mortality but moderate, due to serious imprecision, for mechanical ventilation. The final column provides a plain language summary of the findings.

**Fig. 1:**
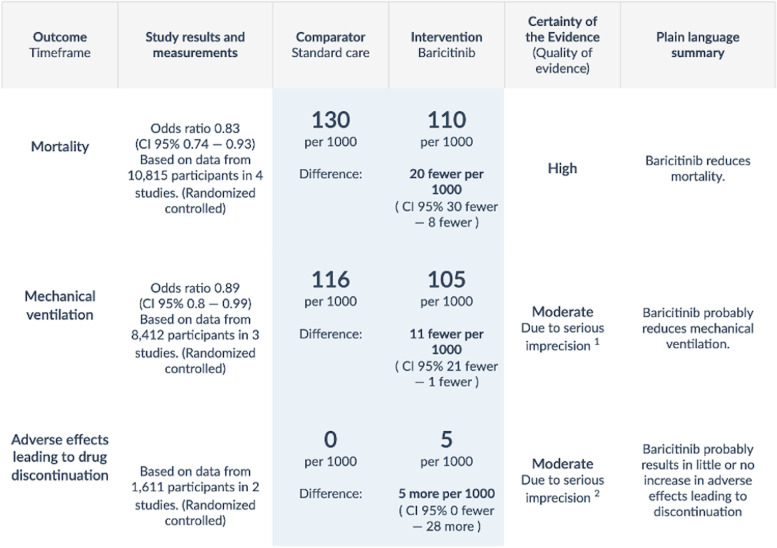
World Health Organization summary of findings table evaluating baricitinib in severe or critical COVID-19 infection [29]
  Clinicians can rely on strong recommendations in trustworthy guidelines while weak recommendations require shared decision-making with patients and/or their representatives. Such conversations involve understanding the values and preferences of patients — either directly or through insights from their representatives — and coming to a decision consistent with those values and preferences [12]. While one might reasonably argue that clinicians should engage in shared decision-making even when recommendations are strong, the time-constrained nature of clinical practice and the resulting necessity to ration time spent on detailed conversations with patients likely makes this unfeasible.
  An example of where further transparency would be desirable is in the 2020 “International evidence-based guidelines on Point of Care Ultrasound (POCUS) for critically ill neonates and children” by the European Society of Paediatric and Neonatal Intensive Care (ESPNIC) [30]. Although the group relies on GRADE, Quaker, RAND/UCLA, and AGREE methods, there is no presentation or discussion of the evidence used to inform the recommendations. Authors report that 28 of their 39 recommendations were based on moderate quality evidence, yet we see only seven randomized trials cited, all of which address POCUS for central catheterization. The presentation makes it impossible to ascertain the true quality of evidence and the magnitude of the benefit of using POCUS.
6. *Might conflicts of interest influence the recommendations? If yes, were they managed?*
  “A conflict of interest exists when a past, current, or expected interest creates a significant risk of inappropriately influencing an individual’s judgment, decision, or action when carrying out a specific duty” [31]. Conflicts of interests are common: a 2019 systematic review found 45% of guidelines had a reported financial conflict, and 32% of authors had undisclosed financial conflicts [32]. Akl et al. (2022) propose a framework to categorize interests, which can be classified as individual (direct financial benefit, benefit through professional status, intellectual and personal) or related to institutional affiliation (direct financial benefit to the institution, benefit through increasing services provided by the institution, and nonfinancial) [31].
  Readers of guidelines may overlook the importance of financial, professional, and intellectual COI: indeed, judging whether COI influence the trustworthiness of a guideline can be challenging. Nevertheless, this assessment plays a key role in determining the credibility of a guideline.
  Although we and other critics of guidelines frequently highlight the need to consider conflicts, the extent to which they actually influence recommendations remains uncertain: A 2020 systematic review of studies evaluating the relative risk of conflicts of interest being associated with favorable recommendations in guidelines was 1.26 (95% confidence interval 0.93–1.69) [33], a confidence interval that includes conflict of interest reducing the likelihood of favorable recommendations. The evidence supporting intellectual conflicts of interest as problematic is also limited and largely rests on a review of breast cancer screening guidelines reported the recommendation of routine screening was increased by an odds of 6.05 (95% confidence interval from 0.57 to infinity, *p* = 0.1) with the presence of radiologists on the guideline, and was associated with the number of recent breast cancer publications by the lead author (*p* = 0.02) [34].

### Applicability

After addressing the six questions and determining that a guideline is trustworthy, clinicians still need to assess whether the recommendations are applicable to their clinical practice. Recommendations from a guideline will be specific to a population and setting. Clinicians should assess the extent to which their patients and setting match those of the recommendations.

The Australian and New Zealand Living Clinical Guidelines for Stroke [35] made a strong recommendation that states that “for patients with potentially disabling ischaemic stroke within 4.5 h of onset who meet specific eligibility criteria, intravenous thrombolysis should be administered as early as possible after stroke onset”. Clinicians who frequently see patients in a time frame slightly longer than the threshold (e.g., 5 h) would have to ponder the implications of the recommendations for these patients.

In the same guideline, the panel strongly recommends that “all stroke patients should be admitted to hospital and be treated in a stroke unit with an interdisciplinary team” [35]. This recommendation is applicable to a clinician working at a tertiary hospital. For a clinician working in an emergency care unit in a rural area or in a low-income country, an interdisciplinary team is unlikely to be available.

In summary, clinicians must evaluate the trustworthiness of a guideline, understand the transparent evidence summary that trustworthy guidelines will offer, and judge the applicability of trustworthy recommendations to their patients and settings. Consideration of the circumstances and values and preferences of patients will be crucial for all weak or conditional recommendations.

## Conclusion

In considering whether to attend to a particular guideline, clinicians should ask themselves six questions — clarity of the recommendation; consideration of all available therapeutic, diagnostic, or prognostic options; consideration of all patient-important outcomes; recommendation should be based on an up-to-date systematic review; strength of the recommendation should be compatible with the certainty of the evidence; and conflicts of interest. Finally, if a guideline is judged credible, clinicians must then assess whether it is applicable to a patient and clinical setting.

## Data Availability

Not applicable.

## Abbreviations

AGREE: Appraisal of Guidelines for Research and Evaluation
ARDS: Acute respiratory distress syndrome
CI: Confidence interval
COI: Conflicts of interests
ESC: European Society of Cardiology
ESPNIC: European Society of Paediatric and Neonatal Intensive Care
GRADE: Grading of Recommendations, Assessment, Development, and Evaluations
HFpEF: Heart failure with preserved ejection fraction
ICU: Intensive care unit
NICE: National Institute for Health and Care Excellence
POCUS: Point of Care Ultrasound
SGLT2: Sodium-glucose cotransporter-2
SoF: Summary-of-findings
VAP: Ventilator-associated pneumonia
